# Enhancing physical characteristics and antibacterial efficacy of chitosan through investigation of microwave-assisted chemically formulated chitosan-coated ZnO and chitosan/ZnO physical composite

**DOI:** 10.1038/s41598-024-58862-6

**Published:** 2024-04-23

**Authors:** Sara A. Ali, E. S. Ali, G. Hamdy, Mona Shaban E. M. Badawy, Abdallah R. Ismail, Inas. A. El-Sabbagh, Magda M. El-Fass, Moataz A. Elsawy

**Affiliations:** 1 Chemistry Department, Faculty of Science, Al-Azhar University Girls, Nasr City, Cairo Egypt; 2 Polymer Laboratory, Petrochemical Department, Egyptian Petroleum Research Institute, Nasr City, 11727 Cairo Egypt; 3 Al-Azhar Technology Incubator (ATI), Nasr City, Cairo Egypt; 4 Department of Microbiology and Immunology, Faculty of Pharmacy (Girls), Al-Azhar University, Nasr City, Cairo Egypt; 5 Department of Processes Design and Development, Egyptian Petroleum Research Institute (EPRI), Nasr City, 11727 Cairo Egypt

**Keywords:** Chitosan, Zinc oxide nanoparticles, Microwave, Antimicrobial activity, Microbiology, Chemistry, Materials science, Nanoscience and technology

## Abstract

This study investigates the creation and analysis of chitosan-zinc oxide (CS-ZnO) nanocomposites, exploring their effectiveness in inhibiting bacteria. Two synthesis approaches, physical and chemical, were utilized. The CS-ZnO nanocomposites demonstrated strong antibacterial properties, especially against *Staphylococcus aureus*, a Gram-positive bacterium. Chemically synthesized nanocomposites (CZ10 and CZ100) exhibited larger inhibition zones (16.4 mm and 18.7 mm) compared to physically prepared CS-Z5 and CS-Z20 (12.2 mm and 13.8 mm) against *Staphylococcus aureus*. Moreover, CZ nanocomposites displayed enhanced thermal stability, with decomposition temperatures of 281°C and 290°C, surpassing CS-Z5 and CS-Z20 (260°C and 258°C). The residual mass percentages at 800°C were significantly higher for CZ10 and CZ100 (58% and 61%) than for CS-Z5 and CS-Z20 (36% and 34%). UV–Visible spectroscopy revealed reduced band gaps in the CS-ZnO nanocomposites, indicating improved light absorption. Transmission electron microscopy (TEM) confirmed uniform dispersion of ZnO nanoparticles within the chitosan matrix. In conclusion, this research underscores the impressive antimicrobial potential of CS-ZnO nanocomposites, especially against Gram-positive bacteria, and highlights their enhanced thermal stability. These findings hold promise for diverse applications in industries such as medicine, pharmaceuticals, and materials science, contributing to the development of sustainable materials with robust antimicrobial properties.

## Introduction

Green chemistry has emerged as a promising approach for addressing the pressing need for sustainable development. The use of biopolymers derived from renewable resources is a key area of focus in this field, owing to their biodegradability, abundance, and unique properties^1,2^. Among the various biopolymers, polysaccharides have been extensively studied for their potential in creating functional materials. In particular, cellulose, chitin, and starch are commonly investigated for their applications in various industries, including food, medical, and pharmaceutical sectors^3,4^. Polysaccharides are of particular interest in the medical field due to their biocompatibility and biodegradability^5^, which make them ideal candidates for drug delivery systems, wound healing, and tissue engineering^6^.

Chitosan (CS), the second-largest renewable biopolymer after cellulose, has attracted considerable attention in various fields due to its unique properties, such as biocompatibility, nontoxicity, and antimicrobial activity^7–13^. However, its poor mechanical and electrical properties limit its potential applications^14^. Therefore, incorporating nanofillers is an effective approach to improving chitosan's physical and mechanical properties^15^. Additionally, chitosan's numerous amino and hydroxyl groups make it an excellent adsorbent for heavy metal ions and dye uptake^16,17^. In recent years, nanomaterials have emerged as promising additives for improving the properties of biopolymers^18^. Zinc oxide nanoparticles (ZnO NPs) are widely used in various fields due to their broad-spectrum antimicrobial activities^19,20^, biocompatibility, and low production cost. Chitosan-coated ZnO nanocomposites have been extensively reported as a key material in various applications, such as food packaging^21^, coating on textile fabric, and dye removal^22–24^, based on their antimicrobial^25,26^, antibiofouling, and photocatalytic activity^18,27^.

This study aims to develop a chitosan-coated zinc oxide (CS-ZnO) nanocomposite using chemical methods [CS-Z and CZ] compared to the physical method preparation and investigate its antibacterial activity against various microorganisms, including Gram-positive bacteria, Gram-negative bacteria, yeast, and filamentous fungus. The nanocomposite was thoroughly characterized using X-ray diffraction spectroscopy and Fourier transforms infrared spectroscopy. The results of this study demonstrate that the CS-ZnO nanocomposite exhibits excellent antibacterial activity against various microorganisms, including both Gram-positive and Gram-negative bacteria. Moreover, the study provides insights into the potential applications of the CS-ZnO nanocomposite in various fields, such as medical, pharmaceutical, and industrial processes.

The utilization of microwave technology in the chemical-mediated synthesis method offers notable advantages in terms of energy efficiency and reaction kinetics. By harnessing microwave irradiation, we achieved rapid heating of the reaction mixture, leading to shortened reaction times and enhanced product yields. Moreover, the precise control over reaction conditions enabled by microwave technology minimizes the generation of unwanted byproducts and waste, aligning with the principles of green chemistry. Additionally, the physical-mediated synthesis method, employing solution-based techniques and sonication, offers distinct benefits in terms of sustainability. This approach minimizes the use of harsh chemicals and promotes eco-friendly processing by relying on physical mechanisms for nanoparticle dispersion within the polymer matrix. The energy-efficient disruption of particle agglomerates through sonication ensures uniform dispersion, contributing to the overall greenness of our nanocomposite fabrication process.

Furthermore, the use of biopolymers in material development, particularly in the medical field, holds great promise for sustainable development. The development of chitosan-based nanocomposites, such as the CS-ZnO nanocomposite described in this study, can further expand the potential applications of biopolymers in various fields. The results of this study contribute to the ongoing efforts to develop green and sustainable materials that can address the environmental and health challenges facing the world today.

## Experimental

### Materials

Chitosan with a degree of deacetylation of 85% and low molecular weight, extracted from crab shells, was procured from G.T.C Bio Corporation (Hong Kong, China). Glacial acetic acid and zinc oxide nanopowder were sourced from Sigma Aldrich, USA. Distilled water was employed in the preparation of all solutions. All chemicals were used as received and met analytical grade standards.

### Preparation of CS/ZnO nanocomposite physically

For the synthesis procedure, approximately 1 g of chitosan powder was dissolved in 100 ml of 1% acetic acid at room temperature. The solution of dissolved chitosan was then combined with solutions containing varying quantities of zinc oxide nanoparticles (5, 10, 15, and 20 wt%), designated as CS-Z 5, CS-Z 10, CS-Z 15, and CS-Z 20. These mixtures were sonicated for 15 min in 100 ml of distilled water. To this solution, 1.0 M NaOH was added gradually while vigorously stirring until the pH reached 10. The solution was allowed to stand overnight. The resulting solution, containing CS-ZnO nanocomposites, underwent multiple washes with distilled water to eliminate any remaining unreacted materials. Subsequently, the solution was dried in a vacuum oven for 24 h.

### Preparation of CS/ZnO nanocomposite chemically

**Scheme 1:**
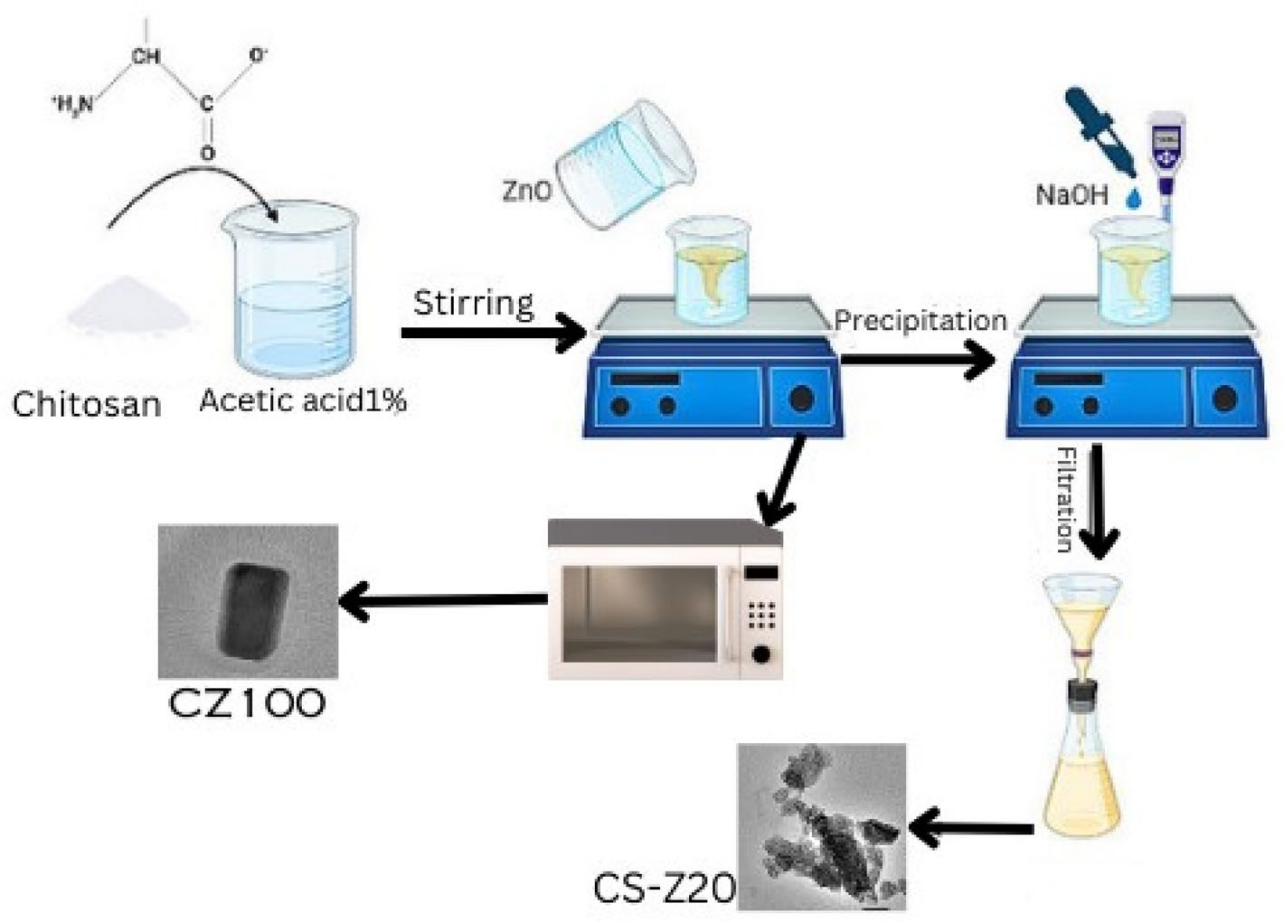
Physical and chemical preparation of Chitosan-Zinc Oxide nanocomposite.

The preparation of CS-ZnO nanocomposites was carried out using a chemical method following the procedure outlined in the referenced literature^18^. Initially, 1 g of chitosan powder was dissolved in 100 ml of 1% acetic acid at room temperature. In a separate step, a solution containing 1 g of zinc oxide was dispersed in 100 ml of distilled water through sonication. This solution was then combined with the previously mentioned chitosan-acetic acid solution in a 500 ml quick-fit bottomed round flask. Subsequently, the assembled flask was placed within a microwave oven and subjected to specific time intervals (3, 5, 7, and 10 min) and power levels (80, 240, 560, and 800 W), denoted as CZ10, CZ30, CZ70, and CZ100. The resulting nanocomposites were subjected to centrifugation and multiple washes using distilled water to eliminate any residual reactants and obtain the final product. The CS-ZnO nanocomposites were then dried in a vacuum oven until a constant weight was achieved (Scheme 1).

### Characterization techniques

The nanocomposites underwent characterization through several techniques. X-ray diffraction (XRD) measurements were performed on a Philip’s X-ray diffractometer PW1390 with Ni-filtered Cu Kα radiation at generator voltage of 4 kV and wavelength of 0.154 nm at room temperature. The diffraction angle, 2θ, was scanned at a rate of 2°min^−1^. Fourier transform infrared (FTIR) spectroscopy was carried out using a JASCO FTIR instrument at Sapala Organics Private Ltd., Hyderabad. The measurements were performed on KBr pellets, covering a spectral range from 400 to 4000 cm^−1^. Thermogravimetric analysis (TGA) was carried out using a thermogravimetric analyzer model SDT Q600 V20.9 build 20. Each sample, weighing approximately 10 mg, was placed in an alumina crucible. The analysis involved subjecting the samples to a temperature range spanning from ambient temperature to 700 °C, with a heating rate of 10 °C/min. The UV–Vis absorption spectra of CS, ZnO NPs and CS/ZnO nanocomposite were obtained by V-750 UV–Vis spectrophotometer, the wavelength of incident ray was selected in the range of 300–600 nm. The morphology of samples has been investigated by transmission electron microscopy (TEM) JEOL (JEM-2100 PLUS, Japan) at an accelerating voltage of 200kV.

### Antimicrobial assay

#### Well diffusion method

The synthesized nanocomposites were evaluated for their anti-microbial activity using the agar diffusion technique^19^. The tested samples evaluated against, Gram + ve bacteria (*Bacillus subtilis* ATCC 6633, *Staphylococcus aureus* ATCC 35,556), Gram -ve bacteria (*Escherichia coli* ATCC 23,282 and *Pseudomonas putida* ATCC 10,145), Yeast (*Candida albicans* IMRU 3669) and Filamentous Fungus (*Aspergillus niger* ATCC 16,404). The bacteria and yeast were grown on nutrient agar while the fungus was grown on Potato Dextrose agar medium. The tested samples were evaluated in the concentration of 5000 ppm. The positive control was *Streptomycin* for bacteria and *Metronidazole* for yeast and fungus.

#### Determination of minimum inhibitory concentration (MIC), minimum bactericidal concentration (MBC), and minimum fungicidal concentration (MFC)

MIC of the prepared compounds that revealed antimicrobial activity against all tested microbes was measured. Broth microdilution method for determining MIC. The nanocomposites are prepared in acetic acid 1% (v/v) solution and is added to each well by using sterile micropipettes in two-fold dilutions to Nutrient broth medium containing 1 × 10^6^ bacterial suspension and kept at 37 ºC for one day. The MIC was calculated using the sample turbidity to determine the lowest concentration of nanocomposite that inhibits 90% of microbial growth. The experiment was repeated three times to ensure that the results were consistent for all of the microorganisms tested^19^. 0.1 ml of each lowest concentration that inhibited the microbial growth were allowed to grow on nutrient agar medium plates to detect MBC and MFC.

## Results and discussion

### X-ray diffraction (XRD) pattern of nanocomposites

**Figure 1:**
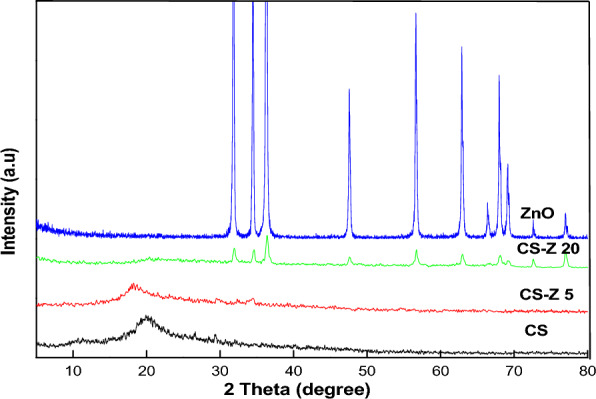
XRD pattern of physically prepared CS-ZnO nanocomposites.

**Figure 2:**
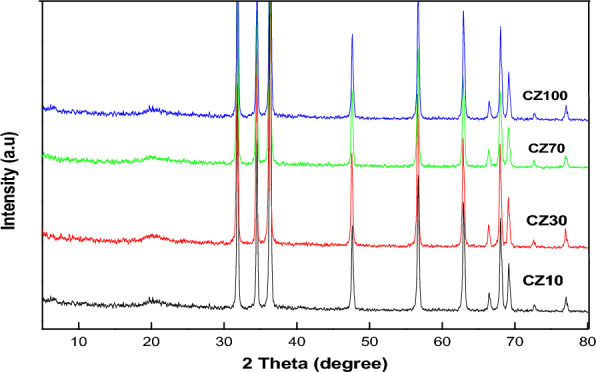
XRD pattern of chemically prepared CS-ZnO nanocomposites.

Figure 1. illustrates the X-ray diffraction (XRD) pattern of CS, ZnO, and physically prepared CS/ZnO nanocomposites. In this depiction, CS exhibits a broad peak at 20.02°, indicative of its semi crystalline nature^28–30^. The diffraction peaks of ZnO nanoparticles (NPs) are notably sharp, aligning with angles of 31.79°, 34.44°, 36.28°, 47.58°, 56.63°, 62.89°, 66.41°, 67.97°, 69.13°, 72.58°, and 76.99°. These patterns correspond to the standard hexagonal phase of ZnO (JCPDS card no. 36-1451)^31–33^. Moving to the CS-ZnO nanocomposites, specifically CS-Z 5, CS-Z 10, CS-Z 15, and CS-Z 20, distinct XRD patterns are observed at different concentrations of ZnO. These patterns retain characteristic peaks of both CS and ZnO NPs, albeit with alterations in intensity and precision. It's noteworthy that the XRD profile of the ZnO-CS nanocomposites underscores the presence of significant peaks related to both CS and ZnO NPs. However, in comparison to the pure CS and ZnO NPs, the intensity of the CS-ZnO nanocomposite peaks is diminished. This could potentially be attributed to interactions between the functional components of CS and ZnO NPs^34^.Fig. 2. portrays the X-ray diffraction (XRD) patterns of nanocomposites prepared via a chemical method utilizing a microwave reactor (CZ10, CZ30, CZ70, and CZ100). These patterns reveal the presence of characteristic peaks associated with both CS and ZnO NPs, exhibiting alterations in both sharpness and intensity. This phenomenon can be interpreted as evidence of a complexation reaction occurring between CS and the surface of ZnO NPs^35^.

### FT-IR spectroscopy

**Figure 3:**
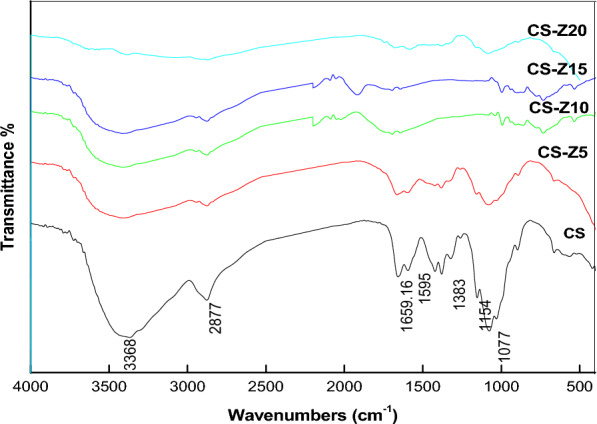
FTIR pattern of physically prepared CS-ZnO nanocomposites.

The FT-IR spectra of CS and physically prepared CS-Z nanocomposites are depicted in Fig. 3. In the infrared spectrum of CS, the characteristic peak at 3368 cm^-1^ can be attributed to the stretching vibrations of amine –NH_2_ and hydroxyl –OH groups. Peaks at 2877, 1659, and 1595 cm^−1^ correspond to –CH stretching vibration, –C = O (amide I) stretching vibration, and –N–H bending (amide II), respectively. The peak at 1380 cm^−1^ is attributed to the stretching of (CH_2_–OH). Peaks at 1154 and 1077 cm^-1^ are assigned to the β (1–4) glycosidic bond and the stretching vibration of C–O–C, respectively.

In the CS-ZnO nanocomposite, the characteristic peak related to the stretching vibration of –NH and –OH groups in CS shifted to a higher wavenumber, specifically 3416 cm^−1^. Similarly, the peak associated with the bending vibration of the –NH group present in CS shifted to a lower wavenumber of 1646 cm^−1^ in the CS-ZnO nanocomposite. Similar phenomena were also observed with the peaks of C–O, 3′-OH, and 5′-OH groups. These shifts were attributed to the formation of hydrogen bonds between ZnO and CS^34^.

In comparison to the IR spectra of pure chitosan film, the spectra of chitosan-ZnO nanocomposites doped with varying concentrations of ZnO NPs, specifically CS-Z 5 and CS-Z 20, exhibited notable shifts in the positions of bands towards both lower and higher wavenumber regions. The width of the bands corresponding to –NH2 and –OH groups is visibly correlated with the quantity of ZnO particles within the chitosan matrix. Furthermore, peaks falling within the 500–600 cm^−1^ range were attributed to the presence of metal oxygen (Zn–O) bonds^36^.

**Figure 4:**
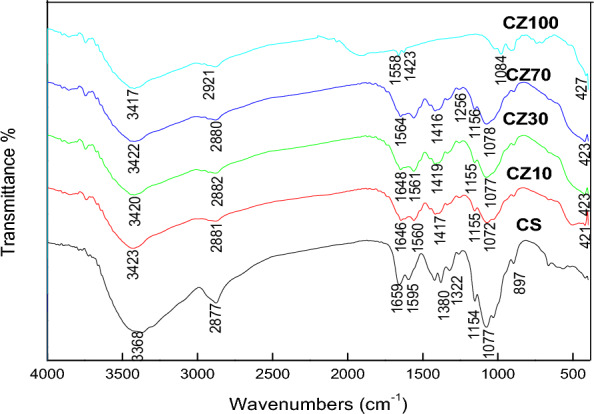
FTIR pattern of chemically prepared CS-ZnO nanocomposites.

Figure 4 displays the FTIR spectrum of CZ70 and CZ100 nanocomposites, which were chemically prepared at different microwave powers, in comparison to the pure CS.

The FTIR spectrum of the chemically synthesized CZ nanocomposite at varying microwave powers is presented in Fig. 4. The acquired IR spectrum exhibited characteristic peaks corresponding to both chitosan and ZnO NPs. Notably; the characteristic bands underwent shifts to lower wavenumbers. For instance, the absorption band at 3368 cm^−1^, associated with the stretching vibrations of hydroxyl, amino, and amide groups, was noticeably shifted to higher wavenumbers. This shift to higher wavenumbers could be attributed to the effective dispersion of Zn NPs within the polymer matrix^37^.

### Thermogravimetric analysis (TGA)

**Figure 5:**
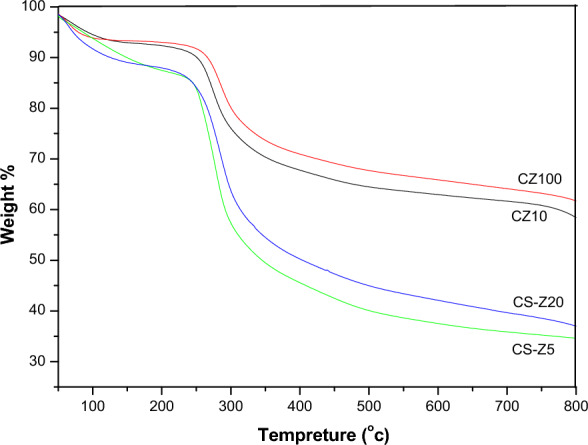
TGA curves of CS/ZnO nanocomposites which prepared by two methods.

The thermal behaviour of both physically and chemically prepared CS/ZnO nanocomposites was subject to thorough investigation using thermogravimetric analysis (TGA), as illustrated in Fig. 5. The TGA results unveiled a striking contrast in thermal stability between the nanocomposites obtained through distinct preparation methods. Remarkably, the chemically prepared CS/ZnO nanocomposites, namely CZ10 and CZ100, exhibited a substantial advancement in thermal stability when juxtaposed with their physically prepared counterparts, CS-Z5 and CS-Z20. The decomposition temperatures of the former, clocking in at 281 °C and 290 °C, noticeably surpassed those of CS-Z5 and CS-Z20, which registered at 260 °C and 258 °C, respectively. This discernible disparity accentuates the efficacy of the chemical preparation approach in enhancing the thermal resilience of the resulting nanocomposites^38^.

The pivotal temperatures at which the foremost decomposition stages occurred showcased a distinct divergence as well. For the CS-Z nanocomposites, this pivotal temperature was around 240 °C, while the CZ nanocomposites demonstrated a higher pivotal temperature of approximately 285 °C. Such findings corroborate the superior thermal stability conferred by the chemical synthesis route, specifically in the CZ nanocomposites.

Further insight was gleaned from the initial decomposition occurring at approximately 100 °C. This initial stage, attributed to the expulsion of water, was exhibited by both the pristine CS and the CS/ZnO nanocomposites.

At the elevated temperature of 800 °C, the residual mass percentages stemming from the decomposition process were notably different among the various nanocomposites. The CS-Zn5 and CS-Zn20 nanocomposites retained 36% and 34% residual mass, respectively. In stark contrast, the CZ10 and CZ100 nanocomposites displayed substantially higher residual mass percentages, reaching 58% and 61%, respectively.

These empirical findings collectively signify the tangible advantage in thermal stability exhibited by the CZ nanocomposites, which can be attributed to the existence of robust interactions between the ZnO nanomaterials and the chitosan matrix. The interplay of chemical synthesis and enhanced interaction mechanisms contributes to the discernible improvement in the thermostability of CZ nanocomposites over their physically prepared counterparts^11^.

### UV–visible spectroscopy

**Figure 6:**
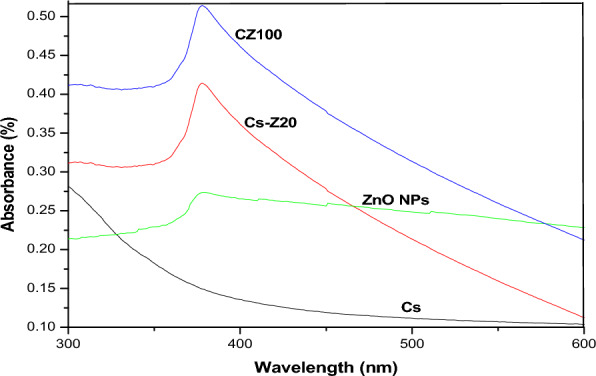
UV–Visible spectra of Cs, ZnO NPs, CZ and Cs-Z nanocomposites.

The UV–Vis absorption spectra of the pure CS, CS-ZnO nanocomposites hybrid and ZnO-NPs are shown in Fig. 6. Figure 6 depicts that in the case of pure CS no UV absorption is observed, because the structure of CS molecules lacks conjugated double bonds^39^. ZnO can be identified as n-type semiconductor with a wide band gap (3.37) eV. Owing to the electron transitions from the valence band to the conduction band, ZnO NPs reveals significant absorption peak between the range of 350–380 nm wavelength^40^. It is interesting to note that the CZ nanocomposites sample demonstrates a sharp absorption peak appeared at around 378, the change in the characteristics of absorption peak can be corresponded to the interactions of ZnO-NPs and CS polymer chains lead to the absorption peak of CS-ZnO to be increased in the visible light region with high intensity compared to CS-Z and ZnO, NPs which indicates the decrease in band gap^41^.

### Morphological analysis

**Figure 7:**
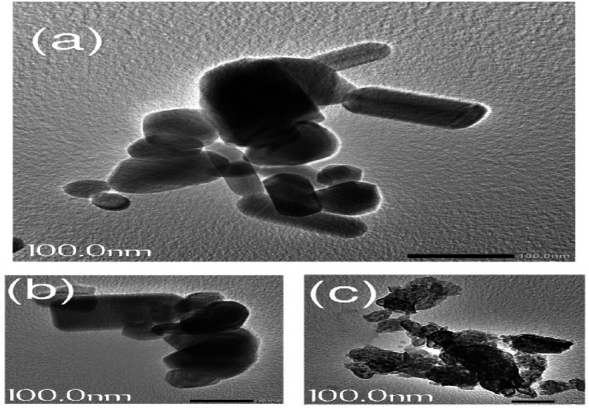
(TEM) images of (**a**) ZnO NPs, (**b**) CZ and (**c**) CS-Z.

Figure 7 illustrates the morphological characterization of pure ZnO NPs and CS-ZnO nanocomposites. Figure 7a demonstrates the approximately hexagonal and spherical form of ZnO NPs^42^. Figure 7b displays CS-Z nanocomposites prepared chemically; the dark areas represent crystalline ZnO nanoparticles whereas the bright spots represent amorphous CS. The morphology of the particles in Fig. 7a shows that the ZnO NPs were evenly dispersed throughout the CS matrix. The sizes of the grains clearly revealed the rod- and cuboid-shaped grains which unmistakably show that the nanosized grains in our chemically created nanocomposites CZ were present. Physically produced CS-Z nanocomposites are shown in Fig. 7C. The aggregation of ZnO NPs into CS matrix led to the confirmation of amorphous background nanoparticles in the CS-ZnO hybrid^11^. The outcomes of the FTIR and XRD investigations were inagreement with the conclusions reached by the electron microscopy evaluations.

### Evaluation of antimicrobial activity

#### Detection of antimicrobial activity of the prepared compounds

**Figure 8:**
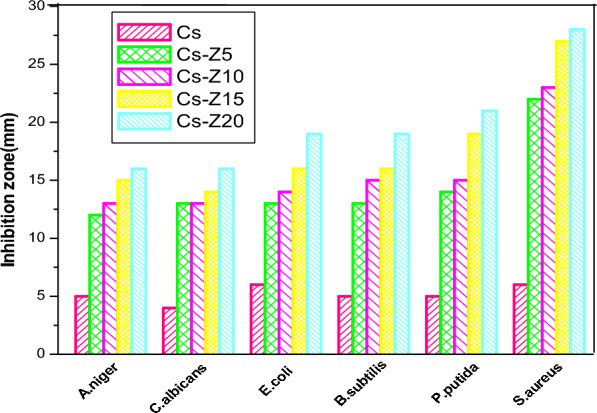
Antimicrobial activity of physically prepared CS-ZnO nanocomposites.

**Figure 9:**
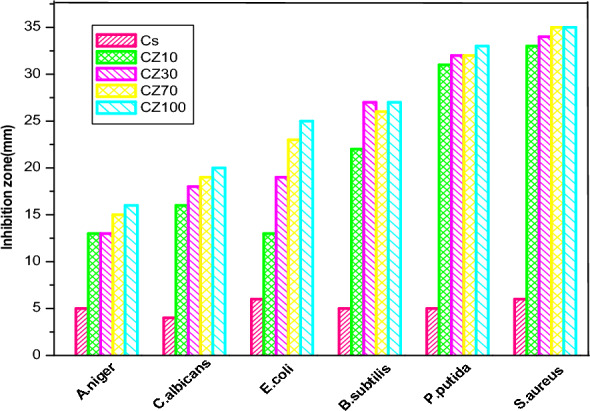
Antimicrobial activity of chemically prepared CS-ZnO nanocomposites.

The antimicrobial effectiveness of both chitosan (CS) and CS-ZnO nanocomposites is visually depicted in Figs. 8 and 9, respectively. The assessment encompassed a variety of microorganisms, namely Gram-positive bacteria (*Bacillus subtilis* and *Staphylococcus aureus*), Gram-negative bacteria (*Escherichia coli* and *Pseudomonas putida*), yeast (*Candida albicans*), and filamentous fungus (*Aspergillus niger*). The evaluation employed the agar diffusion technique, as previously documented^11,29,43–45^.

A distinct pattern becomes evident wherein nanoparticles display substantial zones of inhibition against tested microbes, notably more pronounced at higher concentrations of ZnO in CS-ZnO nanocomposites, as compared to pure CS.

Moreover, the findings demonstrate that *Staphylococcus aureus*, a Gram-positive bacterium, displays heightened vulnerability to CS-ZnO nanocomposites prepared through two distinct methods, in contrast to Gram-negative bacteria, yeast, and fungi. This enhanced sensitivity can be attributed to the efficient and uniform dispersion of ZnO within the chitosan (CS) matrix, particularly prominent in samples synthesized through chemical processes as opposed to physical methods.

The cell membrane of microorganisms is composed of several lipids and protein layers arranged together in a specific arrangement called the bi-layer (or multilayer lipoprotein structure)^46^. The presence of the lipids as building units in the cell membrane gave them their hydrophobic characters^47^. It is clear from the data presented in the figures that all the tested compounds exhibit antimicrobial activities, and the maximum antimicrobial activity was obtained in the case of ZnO 100 this is due to the antimicrobial activity mechanism of nanoparticles differs significantly from that of conventional biocides. The interaction of nanoparticles with the cellular membrane, due to their extremely small size and ability to penetrate it, is the most important contribution of nanoparticles in the biocidal process; after that, the nanoparticles release reactive species by interacting with the cells' inert environment. These reactive species include ionic or free radical threats that assault enzymes and DNA in the cellular nucleus, causing microbial death or growth inhibition^48^.

This discrepancy can potentially be linked to the structural disparities between Gram-positive and Gram-negative bacterial cell walls, making the former more receptive to the antibacterial effects of the composite.

The established antimicrobial activity of chitosan relies on the interaction between the positively charged amino groups (-NH_2_) of chitosan and the negatively charged surfaces of bacterial cells. This interaction can result in the leakage of cellular components, leading to cellular damage^43,49^. Moreover, the overall results collectively indicate that the significant antimicrobial activity of the composite is heavily reliant on the presence of nano ZnO particles embedded within the matrix. The hypothesis is that these ZnO nanoparticles release reactive oxygen species (ROS) and Zn^2+^ ions, which collaboratively target the negatively charged cell walls of bacteria, inducing leakage and eventual cell demise^50,51^. These findings collectively underscore a robust inherent antibacterial potential within the CS -ZnO nanocomposite.

#### Minimum inhibitory concentration and minimum bactericidal and fungicidal concentration

From the data listed in Table 1, it can be observed that the MIC values vary for different compounds and test organisms. The MIC results of the prepared compounds against the tested microbes range from 312 to 1250 ppm, but in case of CZ 70 and CZ 100, the MIC values for *Staphylococcus aureus* is 75, indicating that both chitosan and chitosan/ZnO composite have strong inhibitory effects against this bacterium.

**Table 1:** MIC of the tested compounds.

Tested compounds	*Bacillus subtilis*	*Stapylococcus. aureus*	*Escherichia coli*	*Pseudomonas putida*	*Candida albicans*	*Aspergillus niger*
CZ 70	312 ± 10	75 ± 2	625 ± 10	312 ± 10	625 ± 10	625 ± 10
CZ 100	312 ± 10	75 ± 2	625 ± 10	312 ± 10	625 ± 10	1250 ± 15
Cs-Z15	1250 ± 15	1250 ± 15	625 ± 10	312 ± 10	1250 ± 15	1250 ± 15
Cs-Z15	625 ± 10	625 ± 10	625 ± 10	312 ± 10	1250 ± 15	1250 ± 15

It’s important to note that determining MBC and MFC values involves assessing the viability of microorganisms after exposure to the compounds. It’s found that the tested microbes were totally killed after exposure to the MIC value showed in the table. These findings indicate that chitosan and chitosan/ZnO composite have potential applications as antimicrobial agents, particularly against the tested organisms.

Tables 2 and 3 provide the summary and ANOVA of the results reported in Table 1.

**Table 2:** Summary, count, sum, average, and variance of the MIC results listed in Table (1) including count (The number of observations for each combination of factor levels)), sum (The sum of the observed values for each combination), Average (The mean value for each combination), and Variance (The variance of the observed values for each combination).

Summary	Count	Sum	Average	Variance
312	5	2887	577.4	194,941.3
1250	5	4687	937.4	195,468.8
625	5	4062	812.4	175,906.3
75	3	1950	650	345,625
625	3	1875	625	0
312	3	936	312	0
625	3	3125	1041.667	130,208.3
625	3	3750	1250	0

**Table 3:** Analysis of variance.

Source of Variation	SS	df	MS	F	*p*-value	F crit
Rows	334,083.3	2	167,041.7	2.163811	0.177355	4.45897
Columns	1,647,682	4	411,920.6	5.335903	0.021599	3.837853
Error	617,583.3	8	77,197.92			
Total	2,599,349	14				

SS: Sum of squares, Df: Degree of freedom, MS: Mean sum of squares, F: F-statistic.

*Rows*: The variation between the different rows (factor levels) is evaluated. The F-value of 2.1638 with a *p*-value of 0.1774 suggests that the observed differences between the rows are not statistically significant at the conventional significance level of 0.05. This means that the factor represented by the rows does not have a significant effect on the observed values.

*Columns*: The variation between the different columns (another factor) is evaluated. The F-value of 5.3359 with a p-value of 0.0216 indicates that the observed differences between the columns are statistically significant at the 0.05 significance level. This suggests that the factor represented by the columns has a significant effect on the observed values.

*Error*: This represents the unexplained or residual variation, which is the variation that cannot be attributed to the factors. The Sum of Squares (SS) for Error is 617,583.3333, Degrees of Freedom (df) is 8, and Mean Square (MS) is 77,197.91667.

*Total*: This represents the total variation in the data. The SS for Total is 2,599,348.933, and the df is 14.

## Conclusions

This research demonstrates the successful synthesis of chitosan—zinc oxide (CS-ZnO) nanocomposites through physical and chemical methods. The comprehensive characterization of these nanocomposites using various analytical techniques provides valuable insights into their properties and potential applications.

The X-ray diffraction (XRD) analysis confirmed the presence of both chitosan and ZnO nanoparticles in the nanocomposites, with alterations in peak intensity and precision. Fourier transform infrared (FTIR) spectroscopy revealed shifts in characteristic peaks, indicating interactions between ZnO and chitosan. Thermogravimetric analysis (TGA) demonstrated improved thermal stability in chemically synthesized nanocomposites.

UV–Visible spectroscopy showcased decreased band gaps in CS-ZnO nanocomposites, suggesting enhanced light absorption. Transmission electron microscopy (TEM) revealed the dispersion of ZnO nanoparticles in the chitosan matrix.

The most significant findings pertain to the antibacterial activity of the nanocomposites. Both physical and chemical synthesis methods resulted in nanocomposites with potent antimicrobial properties, with Gram-positive bacteria being more susceptible. This suggests that the CS-ZnO nanocomposites hold promise as effective antibacterial agents, particularly in medical and pharmaceutical applications.

In summary, this study underscores the potential of CS-ZnO nanocomposites in various industries and their contribution to the development of sustainable and versatile materials with robust antimicrobial capabilities. The research paves the way for further exploration and applications of biopolymer-based nanocomposites in addressing critical challenges in health and the environment.

## Data Availability

The datasets used and/or analysed during the current study available from the corresponding author on reasonable request.
